# Polyphyllin I Promoted Melanoma Cells Autophagy and Apoptosis via PI3K/Akt/mTOR Signaling Pathway

**DOI:** 10.1155/2020/5149417

**Published:** 2020-07-17

**Authors:** Jianwen Long, Xianming Pi

**Affiliations:** ^1^Department of Dermatology, Hubei Provincial Hospital of Traditional Chinese Medicine, Hubei Province Academy of Traditional Chinese Medicine, Wuhan 430061, China; ^2^Department of Dermatology, The First Clinical School, Hubei University of Chinese Medicine, Wuhan 430061, China

## Abstract

To investigate whether Polyphyllin I (PPI) might induce the autophagy and apoptosis of melanoma cells by regulating PI3K/Akt/mTOR signal pathway. Melanoma A375 cells were incubated with different concentrations of Polyphyllin I (0, 1.5, 3.0, and 6.0 mg/L) and PI3K/Akt/mTOR signaling pathway activator IGF-1(20 mg/L). CCK-8 assay was utilized to detect cell proliferation; Cell apoptosis and cell cycle were measured by flow cytometry; Western blot was used to examine the expressions of proteins. Immunofluorescence analysis was performed to evaluate autophagy of A375 cells; In addition, xenograft-bearing nude mice were applied to study the role of Polyphyllin I on melanoma development, melanoma cell proliferation, as well as melanoma cell apoptosis in vivo. The outcomes represented that Polyphyllin I promoted A375 cell apoptosis via upregulating Bax level and cleaved caspase-3 level and downregulating Bcl-2 level, inhibited the growth of A375 cells at the G0/G1 phase, and enhanced cell autophagy via regulating the levels of Beclin 1, LC3II, and p62. However, IGF-1 (an activator of PI3K/Akt/mTOR signal pathway) attenuated these changes that Polyphyllin I induced. Furthermore, the xenograft model experiment confirmed that Polyphyllin I treatment suppressed xenograft tumor growth, increased apoptotic index evaluated by the TUNEL method, and reduced the level of Ki67 in tumor tissues in vivo. In conclusion, Polyphyllin I treatment enhanced melanoma cell autophagy and apoptosis, as well as blocked melanoma cell cycle via suppressing PI3K/Akt/mTOR signal pathway. Meanwhile, Polyphyllin I treatment suppressed the development of melanoma in vivo. Therefore, Polyphyllin I possibly is a promising molecular targeted agent used in melanoma therapy.

## 1. Introduction

Melanoma has a distinguishing feature of malignant biological behaviors and drug resistance [1]. In spite of great advances in managing malignant melanoma, particularly, the progression of new targeting treatment and immunization therapy [2], the survival of patients with metastatic melanoma remains poor. Therefore, we should make great efforts to research more effective antitumor medications. Recently, more and more evidence suggests that many Chinese medicinal herbs and their bioactive phytochemicals exhibit potential antitumor functions, providing an alternative therapy strategy for malignant melanoma treatment. For example, increasing studies have confirmed that Luteolin and parthenolide inhibit human melanoma cell proliferation and induce human melanoma cell apoptosis [3, 4]. Polyphyllin I, a bioactive constituent extracted from Paris polyphylla, has extensive bioactivities and pharmaceutic activities. Numerous researches manifested that Polyphyllin I suppressed the proliferation of ovarian cancer cells, osteosarcoma cells, and gastric cancer cells [5–7]. Nevertheless, it is still unknown how Polyphyllin I plays its roles in human malignant melanoma.

Autophagy is a self-degradative cellular process which is essential for balancing sources of energy for vital time [8], as well as is well known to be regulated by PI3K/Akt/mTOR pathway [9]. Phosphorylation of Akt is able to enhance the activity of tuberous sclerosis 1/2, which further contributes to mTOR activation [10, 11]. mTOR activation is involved in negatively regulating autophagy levels by reducing the level of the downstream molecular complex ULK1 [12]. Accumulating researches have confirmed that autophagic activity is changed in various cancers and is considered to be a promising target in cancer treatment [13]. Meanwhile, previous researches have shown that autophagy is interconnected closely with apoptosis by several molecular nodes of crosstalk [14].

We aimed at investigating the effect of Polyphyllin I on melanoma cell proliferation, autophagy, and apoptosis, as well as exploring the underlying mechanism. Furthermore, tumor xenografts were established to evaluate whether Polyphyllin I was capable of suppressing the development of melanoma in vivo.

## 2. Materials and Methods

### 2.1. Cell Lines and Cell Culture

The human melanoma A375 cell line applied in this experiment was purchased from the Cell Bank of the Chinese Academy of Sciences (Shanghai, China). All cells were processed in DMEM supplemented with 10% fetal bovine serum at 37°C in a humidified atmosphere (5% CO2). Dimethyl sulfoxide (DMSO; #12611; CST) was utilized to dissolve Polyphyllin I, and the terminal concentration of dimethyl sulfoxide was less than 0.125% in the experiment. To evaluate whether Polyphyllin I might promote melanoma cells autophagy and apoptosis via PI3K/Akt/mTOR signal pathway, A375 cells were cultured with 0 mg/L, 1.5 mg/L, 3.0 mg/L, 6.0 mg/L Polyphyllin I (Chengdu Must BioTechnology Co., Ltd.), and PI3K/Akt/mTOR pathway activator IGF-1 (20 mg/L, Merck Millipore, Millipore, Billerica, MA, USA) for 48 h, respectively.

### 2.2. CCK-8 Assay

CCK-8 assay was performed to research the function of Polyphyllin I in cell proliferation. After treatment, A375 cells (6 × 10^3^/well) were cultured into 96-well plates for 48 h, and cell viability was calculated in accordance with the protocol. The absorbance at 450 nm was detected to assess cell growth rates.

### 2.3. Cell Invasion and Migration Assay

Wound healing assays were applied when the cells after treatment nearly achieved 90% confluence in six-well plates. A scratch was created using a 100 *μ*L pipette tip in confluent monolayers. At 0 h and 24 h, the plates were assessed and imaged with a microscope. For assessing cell invasion, A375 cells after treatment were seeded onto the upper chamber of Transwell for the invasion assays. DMEM containing 10% FBS was then added into the lower chamber of Transwell. After 24 hours at 37°C in a humidified incubator with 5% CO2, cells in upper chambers were removed, then the penetrated cells were fixed with formaldehyde and stained with crystal violet. The numbers of invaded cells were calculated using a light microscope.

### 2.4. Flow Cytometric Analysis

A375 cells were treated with different concentrations of Polyphyllin I for 48 h, and then cells were digested into single cells using EDTA-free trypsin. Lastly, the cells were stained with the Annexin V-FITC and PI solution (Vazyme, Nanjing, China) at 4°C. Cell apoptosis was calculated after 10-15 min by flow cytometry. For detecting cell cycle, each group of A375 cells was harvested by centrifugation, washed, and resuspended with PBS solution. Subsequently, A375 cells were incubated with propidium iodide (PI) (Vazyme, Nanjing, China) for 30 minutes in the dark. At last, cell cycle status was assessed with flow cytometry.

### 2.5. Immunofluorescence Analysis

After treatment, A375 cells were washed with PBS, fixed with 4% paraformaldehyde for 20-30 min, and then permeabilized with 0.2% Triton X-100 for 15 min. After blocking, the slides were incubated with primary antibodies LC3A/B (12741, CST) and Alexa Fluor 488-conjugated secondary antibody according to the protocol, and then the slides were counterstained with DAPI. Finally, after the slides were washed with PBS three times, images were acquired using a laser scanning microscope.

### 2.6. Western Blotting

A375 cells were inoculated with Polyphyllin I for 48 h. The protein content was quantified by a BCA Protein Assay (Beyotime, China). Cell protein lysates were electrophoresed through 10% SDS-PAGE and transferred to PVDF membranes. The membranes were blocked with 5% fat-free milk. Subsequently, the primary antibody was applied for 12 hours incubation at 4°C. The membranes were washed twice and visualized with HRP-conjugated secondary antibodies for 2 h. The protein levels were evaluated by the ECL detection kit. Primary antibodies included PI3K (4249, CST), p-PI3K (Ser1070) (bs-6417R, Bioss Inc.), p-AKT (Ser473) (4060, CST), AKT (2920, CST), mTOR (2972, CST), p-mTOR (Ser2448) (2971, CST), LC3A/B (12741, CST), Beclin 1 (3495, CST), P62 (16177, CST), Bcl-2 (15071, CST), Bax (5023, CST), cleaved caspase-3 (9661, CST), and *β*-actin (3700, CST).

### 2.7. Xenograft Tumor

The research was performed with the approval of the Ethics Committee of Hubei provincial hospital of traditional Chinese medicine. Under specific pathogen-free conditions, four-week-old male BALB/c nude mice, from the Shanghai Laboratory Animal Center, were adopted to establish a xenograft model. 16 nude mice were randomly separated into a control group (8 mice) and Polyphyllin I group (8 mice). A375 cells (2 × 10^6^) were inoculated into nude mice. Polyphyllin I (5 mg/kg) (PPI group) or a corresponding volume of vehicle (control group) was delivered by intraperitoneal injection once a day starting 7 days after tumor inoculation. After 35 days of treatment, mice were euthanized by cervical dislocation, and tumors were dissected and weighted. The tumor volume was measured according to the formula: volume (mm^3^) = (short diameter)^2^ × (long diameter)/2. Four-micron-thick serial sections of formalin-fixed and paraffin-embedded xenograft tissues were applied to detect cell apoptosis and proliferation by TUNEL and Ki67 staining.

### 2.8. Statistical Analysis

All analyses were performed using SPSS version 15.0. Data were recorded as mean ± SD. The statistical significance was determined by one-way ANOVA to compare three or more groups or by Student's *t*-test for the comparison between two groups. *P* < 0.05 was considered significant.

## 3. Results

### 3.1. Polyphyllin I Treatment Inhibited Melanoma Cells Growth

**Figure 1:**
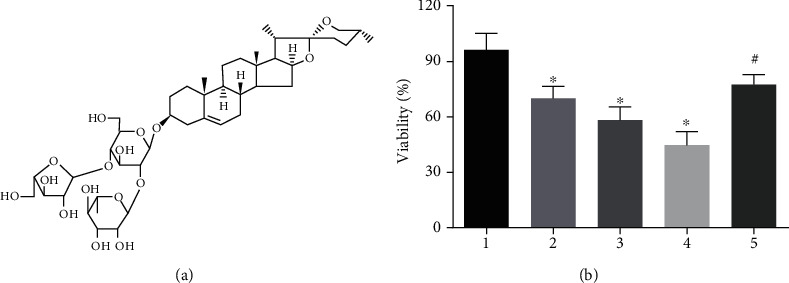
The effect of Polyphyllin I on cell viability. (a) Chemical structure of Polyphyllin I. (b) Polyphyllin I treatment inhibited significantly A375 cell proliferation, but IGF-1 treatment attenuated considerably the inhibitory function of Polyphyllin I in A375 cells growth. ^∗^*P* < 0.05 in comparison with 0 mg/L PPI group; ^#^*P* < 0.05 in comparison with 6 mg/L PPI group. Note: 1: 0 mg/L PPI group; 2: 1.5 mg/L PPI group; 3: 3.0 mg/L PPI group; 4: 6.0 mg/L PPI group; 5: 6.0 mg/L PPI +20 mg/L IGF-1group.

A375 cells were treated with Polyphyllin I (Figure 1(a)) of indicated doses (0, 1.5, 3.0, and 6.0 mg/L) for 48 h, and then CCK-8 assay was applied to determine cell growth. The results manifested that Polyphyllin I treatment could inhibit A375 cell proliferation in comparison with 0 mg/L PPI group (*P* < 0.05). In addition, IGF-1 enhanced A375 cells proliferation suppressed by Polyphyllin I treatment in comparison with 6 mg/L PPI group (*P* < 0.05), indicating a dependence on PI3K/Akt/mTOR signal pathway for the Polyphyllin I-suppressed cell proliferation in A375 cells (Figure 1(b)).

### 3.2. Polyphyllin I Attenuated the Migration and Invasion of Melanoma A375 Cells

**Figure 2:**
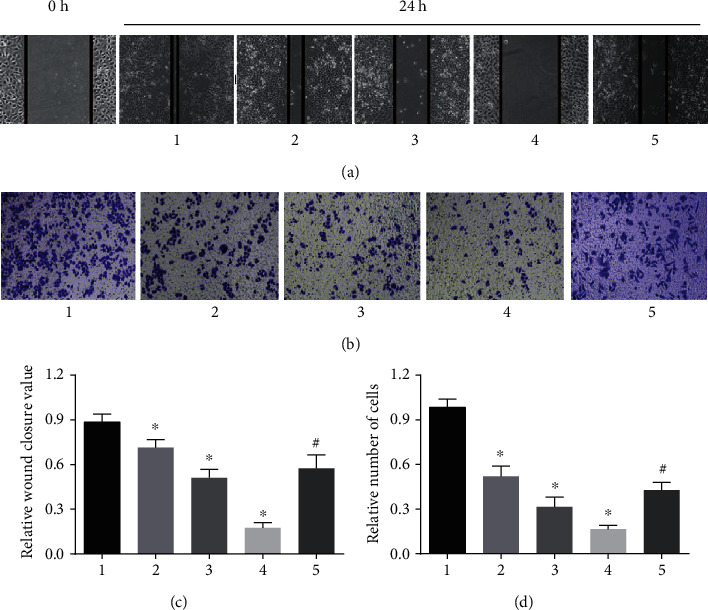
Polyphyllin I suppressed melanoma A375 cell migration and invasion in a dose-dependent manner. (a, c) A375 cell migration as measured by Wound healing assays was inhibited by Polyphyllin I treatment, but IGF-1 treatment mitigated extremely the inhibitory function of Polyphyllin I in A375 cell migratory capability. (b, d) A375 cell invasion as determined by Transwell assays was suppressed distinctly by Polyphyllin I treatment. However, IGF-1 treatment relieved dramatically the inhibitory function of Polyphyllin I in A375 cells invasive capability. ^∗^*P* < 0.05 in comparison with 0 mg/L PPI group; ^#^*P* < 0.05 in comparison with 6 mg/L PPI group. Note: 1: 0 mg/L PPI group; 2: 1.5 mg/L PPI group; 3: 3.0 mg/L PPI group; 4: 6.0 mg/L PPI group; 5: 6.0 mg/L PPI +20 mg/L IGF-1 group.

The migratory and invasive capabilities of melanoma A375 cells were evaluated by wound-healing assay and Transwell assay, respectively. We found that Polyphyllin I alleviated significantly the A375 cells ability to traverse the matrigel in comparison with 0 mg/L Polyphyllin I group (*P* < 0.05). Wound healing assay represented that the treatment with Polyphyllin I significantly attenuated the migration ability of A375 cells in comparison with 0 mg/L Polyphyllin I group (*P* < 0.05). However, IGF-1 treatment relieved dramatically the inhibitory effect of Polyphyllin I on A375 cell migration and invasion. (Figure 2).

### 3.3. Polyphyllin I Treatment Suppressed Cell Cycle Progression of Melanoma Cells

**Figure 3:**
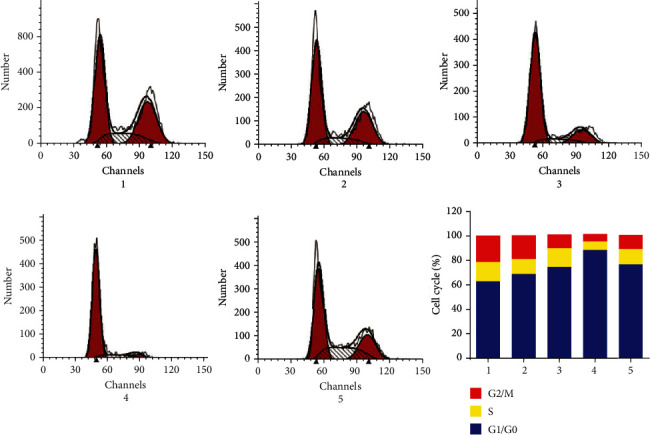
Polyphyllin I treatment suppressed cell cycle progression of melanoma cells. Polyphyllin I triggered cell cycle blockage in A375 melanoma cells in the G0/G1 phase, but IGF-1 attenuated the inhibitory effect of Polyphyllin I on the A375 cell cycle. Note: 1: 0 mg/L PPI group; 2: 1.5 mg/L PPI group; 3: 3.0 mg/L PPI group; 4: 6.0 mg/L PPI group; 5: 6.0 mg/L PPI +20 mg/L IGF-1group.

The effect of Polyphyllin I on the cell cycle progression of A375 cells was determined by flow cytometric analysis. The outcomes revealed that A375 cells treated with 1.5, 3.0, and 6.0 mg/L Polyphyllin I for 48 h were mainly blocked at the G1 phase, and the percentage of G2 phase and S phase manifested a remarkable decrease, as compared to 0 mg/L Polyphyllin I group (*P* < 0.05). However, IGF-1 attenuated the function of Polyphyllin I in G1 phase blockage in A375 cells (Figure 3).

### 3.4. Polyphyllin I Treatment Enhanced the Apoptosis of Melanoma Cells

**Figure 4:**
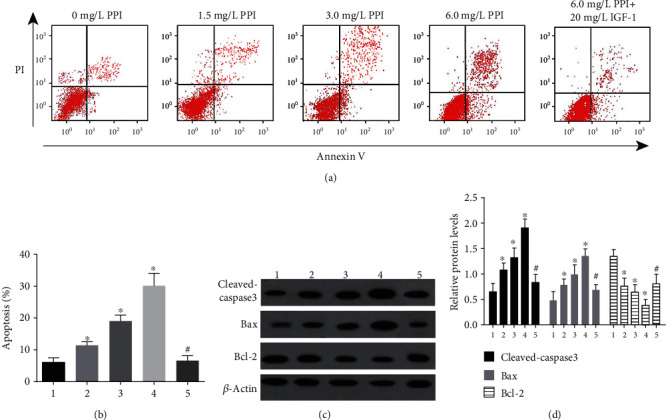
Polyphyllin I treatment affected A375 cells apoptosis in vitro. (a, b) Polyphyllin I treatment enhanced obviously A375 cells apoptosis. (c, d) Western blot assays manifested that the expressions of Bax and cleaved caspase-3 were increased distinctly, but the level of Bcl-2 was significantly decreased in A375 cells after Polyphyllin I treatment in comparison with 0 mg/L Polyphyllin I group. However, IGF-1 treatment alleviated distinctly the above mentioned functions of Polyphyllin I in A375 cells apoptosis. ^∗^*P* < 0.05 in comparison with 0 mg/L PPI group; ^#^*P* < 0.05 in comparison with 6 mg/L PPI group. Note: 1: 0 mg/L PPI group; 2: 1.5 mg/L PPI group; 3: 3.0 mg/L PPI group; 4: 6.0 mg/L PPI group; 5: 6.0 mg/L PPI +20 mg/L IGF-1group.

As a result in Figure 4, Polyphyllin I distinctly elevated the percentage of apoptosis in A375 cells in comparison with 0 mg/L PPI group (*P* < 0.05). However, IGF-1 reversed the promoting effect of Polyphyllin I on the apoptosis of A375 cells. Furthermore, the expressions of apoptosis-associated proteins were measured to understand the molecule mechanisms. As we expected, the levels of Bax and cleaved caspases-3 were significantly elevated, but the level of Bcl-2 was remarkably reduced in comparison with 0 mg/L Polyphyllin I group (*P* < 0.05). However, IGF-1 treatment alleviated distinctly the above-described functions of Polyphyllin I (*P* < 0.05).

### 3.5. Polyphyllin I Treatment Enhanced Melanoma Cells Autophagy

**Figure 5:**
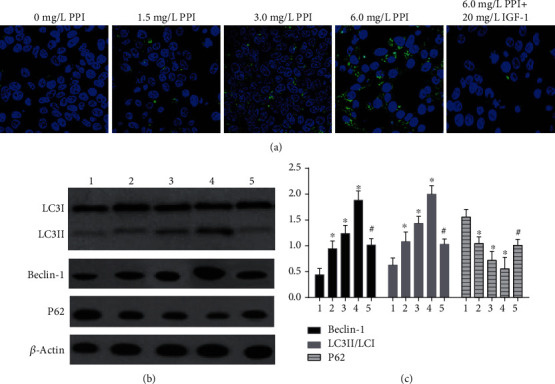
Effects of Polyphyllin I on the autophagy of A375 melanoma cells. (a) LC3 expression was displayed by confocal microscopy; (b, c) The levels of Beclin 1 and LC3II were significantly enhanced, but the expression of P62 was distinctly decreased after Polyphyllin I treatment in A375 cells. IGF-1 treatment attenuated distinctly the promoting functions of Polyphyllin I in A375 cells autophagy. ^∗^*P* < 0.05 in comparison with 0 mg/L PPI group; ^#^*P* < 0.05 in comparison with 6 mg/L PPI group. Note: 1: 0 mg/L PPI group; 2: 1.5 mg/L PPI group; 3: 3.0 mg/L PPI group; 4: 6.0 mg/L PPI group; 5: 6.0 mg/L PPI +20 mg/L IGF-1group.

To investigate whether Polyphyllin I could enhance autophagy in A375 cells, we still investigated the prominent markers of autophagosome formation, such as Beclin 1, LC3, and P62. The expression of LC3 was determined by immunofluorescence staining assay, and the number of LC3 fluorescence dots per cell was distinctly increased in A375 cells 48 h after treatment with Polyphyllin I of indicated concentration as compared to 0 mg/L PPI group (*P* < 0.05). Meanwhile, Western blotting showed that the levels of Beclin 1 and LC3II were significantly enhanced, but the expression of P62 was distinctly decreased 48 h after treatment with Polyphyllin I of different concentrations in A375 cells, as compared to 0 mg/L PPI group (*P* < 0.05). Therefore, we considered that Polyphyllin I was able to promote autophagy of A375 cells. On the other hand, IGF-1 treatment alleviated distinctly these functions of Polyphyllin I in promoting A375 cells autophagy (Figure 5).

### 3.6. Polyphyllin I Treatment Suppressed the Activation of PI3K/Akt/mTOR Signal Pathway

**Figure 6:**
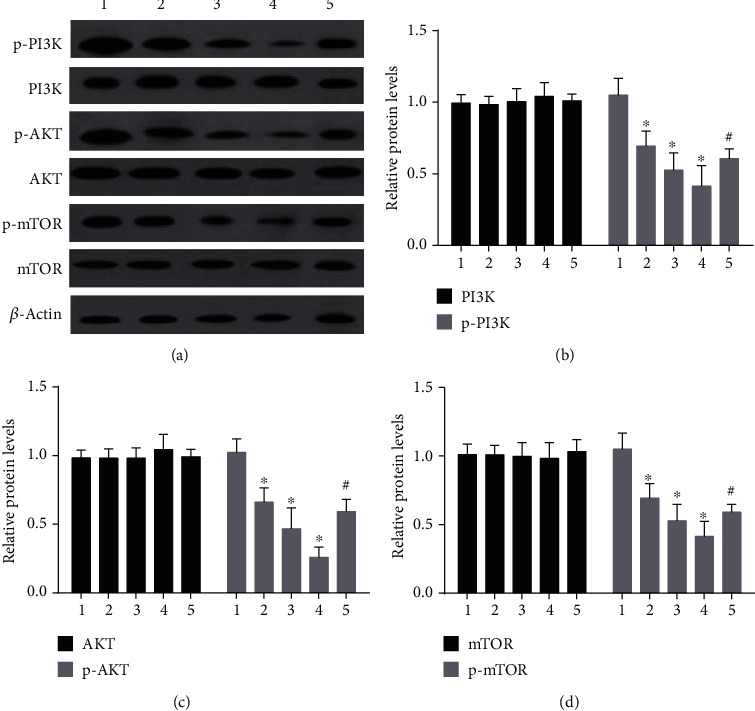
Polyphyllin I inhibited PI3K/AKT/mTOR signaling pathways in A375 melanoma cells. (a) Western blot assay was performed to assess the expressions of proteins related with PI3K/AKT/mTOR signaling pathways. (b–d) Polyphyllin I treatment dramatically attenuated the expressions of p-PI3K, p-AKT, and p-mTOR, but made no difference to the protein expressions of PI3K, AKT, and mTOR. IGF-1 treatment alleviated distinctly the effects of Polyphyllin I on the expressions of p-PI3K, p-AKT, and p-mTOR. ^∗^*P* < 0.05 in comparison with 0 mg/L PPI group; ^#^*P* < 0.05 in comparison with 6 mg/L PPI group. Note: 1: 0 mg/L PPI group; 2: 1.5 mg/L PPI group; 3: 3.0 mg/L PPI group; 4: 6.0 mg/L PPI group; 5: 6.0 mg/L PPI +20 mg/L IGF-1group.

To further confirm that the PI3K/Akt/mTOR signaling pathway was related to Polyphyllin I-induced autophagy and apoptosis. The efficacy of Polyphyllin I on the PI3K/Akt/mTOR signal pathway was evaluated in A375 cells. As represented in Figure 6, the expressions of p-PI3K, p-Akt, and p-mTOR were distinctly decreased in A375 cells after treatment with Polyphyllin I in comparison with 0 mg/L PPI group (*P* < 0.05), but the total protein expressions of PI3K, Akt, and mTOR had no significant change before and after treatment (*P* > 0.05). In addition, IGF-1 treatment attenuated obviously the inhibitory influence of Polyphyllin I on the PI3K/Akt/mTOR signal pathway (Figure 6).

### 3.7. Polyphyllin I Treatment Inhibited A375 Melanoma Cells Xenograft Growth

**Figure 7:**
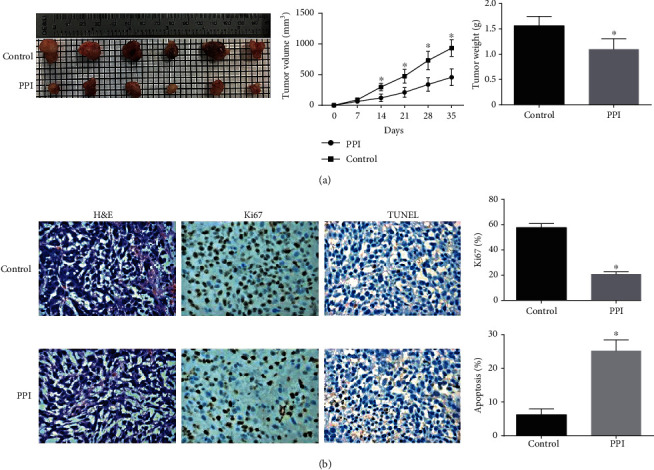
Images of excised tumors from mice at 35 days. (a) The comparison of tumor weight and volume between the control group and the Polyphyllin I group. (b) Representative images of H&E staining and immunohistochemical staining of Ki67 and TUNEL in tumor sections of the two groups. ^∗^*P* < 0.05 in comparison with the control group.

To further identify the functions of Polyphyllin I as mentioned above, animal experiments were conducted to investigate the effect of Polyphyllin I on tumor growth and cell apoptosis in vivo. Xenografts were formed by A375 cells. A remarkable decrease in tumor weight and size was confirmed after treatment with Polyphyllin I in comparison with the control group (Figure 7(a)) (*P* < 0.05). In addition, to research the underlying mechanism of Polyphyllin I on suppressing melanoma, a histological analysis was utilized to detect tumor cell apoptosis and proliferation in vivo. The outcomes manifested that more TUNEL positive cells were found in the xenografts treated with Polyphyllin I in comparison with the control group. Meanwhile, the expression of Ki67 was dramatically reduced in the xenograft model treated with Polyphyllin I in comparison with the control group (Figure 7(b)). In brief, the effect of Polyphyllin I was further identified on inhibiting melanoma growth in vivo.

## 4. Discussion

Apoptosis plays a critical role in the development of melanoma [15]. It represents an attractive therapeutic schedule for treating melanoma to promote melanoma cell apoptosis. As two pivotal factors, Bcl-2 and Bax are conceived to be involved in the control of apoptosis through the mitochondrial pathway [16]. In addition, caspase-3 is regarded as a key protease to coordinate the execution of apoptosis [17]. On the basis of the experimental outcomes, Polyphyllin I distinctly elevated the apoptosis of A375 cells. Similarly to our results, Luo et al. revealed that Polyphyllin I induced hepatocellular carcinoma HepG2 Cells apoptosis via regulating the expressions of Bcl-2, Bax, and cleaved caspase-3 [18]. Therefore, it was verified that Polyphyllin I was able to promote melanoma cell apoptosis by regulating the levels of apoptotic-associated factors.

As we know, autophagy is a catabolic process for the degradation of cytoplasmic constituents in the lysosome and is essential to retain cell metabolism and promote cell survival in stress situations [19]. Beclin 1 is an important factor in the regulation of autophagy and is considered as a tumor inhibitor [20]. LC3 converts to LC3-I under the effect of Atg4, and then LC3-I is further transformed into LC3-II in the presence of Atg5/Atg7/Atg12 [21, 22]. The presence of LC3-II has been considered as a symbol of autophagosome formation [23]. Meanwhile, as a marker of autophagic flux, P62 is reduced in the last phage of autophagy and is negatively correlated with the level of autophagy activity [24]. Tian et al. reported that Polyphyllin I promoted human acute myeloid leukemia cell autophagy by inhibiting JNK and mTOR pathways [25]. Analogously, Polyphyllin I was capable of triggering the autophagy process of melanoma A375 cells by modulating the levels of autophagy-associated factors.

Furthermore, we would like to know how Polyphyllin I regulated melanoma cells apoptosis and autophagy, PI3K/Akt/mTOR signaling pathway activator IGF-1 was used. It was obvious that IGF-1 attenuated the function of Polyphyllin I in promoting melanoma cell apoptosis and autophagy. These results demonstrated that the PI3K/Akt/mTOR signaling pathway potentially was involved in the effects of Polyphyllin I on cell apoptosis and autophagy. Subsequently, we researched the changes of these key proteins associated with PI3K/Akt/mTOR signaling pathway in A375 cells after Polyphyllin I treatment, the results manifested that PI3K/Akt/mTOR signaling pathway was inhibited, the expressions of p-PI3K, p-Akt and p-mTOR was distinctly decreased, but the total protein levels of PI3K, Akt, and mTOR had no significant difference in melanoma A375 cells. To our knowledge, the activated PI3K/Akt/mTOR signaling pathway is able to suppress melanoma cell autophagy and apoptosis [26, 27]. Therefore, we considered that the inhibitory effect of Polyphyllin I on the PI3K/Akt/mTOR signaling pathway played indispensable roles in suppressing melanoma growth.

As manifested in the results, Polyphyllin I was capable of inhibiting the proliferation of melanoma cells by blocking the cell cycle at the G0/G1 stage. Consistently, Zhu et al. released that Polyphyllin I triggered cell cycle arrest and cell apoptosis in human Retinoblastoma Y-79 cells by targeting p53 [28]. Besides, we confirmed that Polyphyllin I treatment significantly attenuated melanoma cell migration and invasion in vitro. At last, the xenograft experiment offered more evidences in the antitumor effect of Polyphyllin I on melanoma. For example, the volume and weight of xenograft were obviously reduced, melanoma cell proliferation was distinctly suppressed and melanoma cell apoptosis was evidently enhanced in tumor xenograft.

All in all, Polyphyllin I induces melanoma cell autophagy and apoptosis, arrest melanoma cells stage at G0/G1 by inhibiting PI3K/Akt/mTOR signal pathway, thus suppressing melanoma progression. Therefore, Polyphyllin I possibly is a promising molecular targeted agent used in melanoma therapy.

## Data Availability

The data used to support the findings of this study are available from the corresponding author upon request.
